# The role of parental education in child disability in China from 1987 to 2006

**DOI:** 10.1371/journal.pone.0186623

**Published:** 2017-10-17

**Authors:** Ping He, Gong Chen, Zhenjie Wang, Chao Guo, Xiaoying Zheng

**Affiliations:** 1 Institute of Population Research, Peking University, Beijing, China; 2 APEC Health Science Academy (HeSAY), Peking University, Beijing, China; Universita degli Studi di Perugia, ITALY

## Abstract

This paper aimed to investigate the role of parental education in child disability in China. We used nationally representative data from China’s National Sample Survey on Disability, iterated twice, in 1987 and 2006, with data of 764,718 children aged 0–14 years. Logit models were used for statistical analysis. Results showed that the prevalence of child disability was significantly associated with each parent’s education. Maternal education was more important than paternal education in child disability in both surveys. The analysis of marginal effect indicated a one-year increase in maternal and paternal schooling led to an average decrease of 0.121% and 0.091% in the probability of child disability in 1987, and 19 years later, these figures had dwindled to 0.091% and 0.072%, respectively.

## Introduction

During recent decades, coordinated international efforts in research have led to substantial reduction in child mortality rates in low- and middle-income nations [1]. These improvements in child survival, however, have coincided with a growing global awareness of the prevalence of child disability [2–4]. Studies on child health status need to take parental socioeconomic status into account since family background of children determines their access to and utilization of health care [5–7]. Numerous studies have investigated this intergenerational relationship [8–10]. Education, as a factor of socioeconomic status, is known to be associated with health [11, 12].

Previous evidence regarding the association of parental education with child health is mixed. On the one hand, a vast majority of studies have addressed the role of maternal education. Since Caldwell’s seminal work [13], it has been maintained that maternal education is a critical determinant of child health [14, 15]. Studies have found that maternal education[16–20], rather than paternal education [21], is associated with child health. On the other hand, other studies have investigated the impact of both parents’ education on child health [22, 23], and compared each parent’s relative importance in child health [24]. Consequently, not only does the education of each parent have a significant impact on child health, but, in some cases, compared to the mother’s schooling, that of the father has an independent [14], equally important [25], or even slightly greater effect [26].

Like other countries, Chinese studies mainly focused on the association between maternal education and child health. According to the Annual Statistics of the National Health and Family Planning Commission, China achieved the MDG target of reducing infant mortality in 2007, well in advance of the target date, and the severe malnutrition rate in children under five reduced from 3.1% in 2000 to 1.4% in 2012. Previous studies have found maternal education is associated with child nutritional status[27, 28]. Up to now, there is only one study investigating the relationship between maternal education and child intellectual disability[29]. We have not found any evidence to study each parent’s role and to compare the relative importance in child disability.

In this study, using data from the two iterations of China’s National Sample Survey on Disability, completed in 1987 and 2006, we aimed to examine: a) whether each parent’s education is associate with child disability; and b) if so, whose education shows a greater role, namely, that of the mother or that of the father. An examination for this study will fill gaps on this issue in China and contribute to international literature in the eastern context.

## Methods

### Data source

Data for this study were obtained from a unique, nationally representative, and large-scale dataset on disabled persons in China. The Chinese government has implemented the National Sample Survey on Disability twice, in 1987 and 2006. The purpose of this survey was to estimate the distribution of individuals with various types and levels of disability at the national and provincial levels; to examine the demographic and socioeconomic situations of households containing persons with disabilities; to identify the causes, timing, and medical treatment of disability; and to document the activities of disabled persons and their participation in social protection programs. The results of these surveys serve as the basis for national and local policies and guidelines regarding persons with disabilities in China[30].

Definitions of all types of disabilities in the 1987 survey were based on the International Classification of Impairments, Disabilities and Handicaps[31], and those of the 2006 survey were based on the International Classification of Functioning, Disability, and Health[32]. The definitions of difference types of disabilities and the survey questions were presented in S1 Table [33].

Multistage, stratified random-cluster sampling, with probability proportional to size, in all provinces of China was used in both surveys. First, children aged 0–6 years were screened for visual, hearing, speech, motor, intellectual or psychiatric disability by physicians or specialists at health clinics or community health stations, and the screening questions were answered by children’s parents. Children aged 7–14 years were interviewed by trained interviewers at the selected households, and the screening questions were answered by children. Afterwards, all suspected children with any type of disability were referred to physicians or pediatricians to make final diagnosis. More details on sample processing and screening were presented in our previous work [33].

Both surveys excluded the institutionalized population. The two studies surveyed 424 counties (3,169 communities) in 1987 and 734 (5,964) in 2006. The sample size was 1,579,316 in 1987 and 2,526,145 in 2006, representing 1.5 and 1.9 per 1,000 non-institutionalized inhabitants of China, respectively [33].

### Participants

The two surveys included socioeconomic indicators of households, main demographic characteristics for each member, and their relationships to the heads of households, which allowed us to match children with their parents. In this study, we selected a subsample of children aged 0–14 years and their parents aged 15–59 years at childbirth. Only children having both parents were enrolled in our study as we need to compare effects of maternal and paternal education on child disability. In total, there were 408,093 and 356,625 observations in 1987 and 2006, respectively.

### Measures

The primary outcome variable was disabled status, i.e., whether a child had any type of disability. The types of child disability included visual, hearing, speech, motor, intellectual and psychiatric disability. To check the robustness of the role of parental education on child disability, other outcome variables included physical and mental disabled status, namely, whether having a physical disability (visual, hearing and motor disability) and mental disability (intellectual and psychiatric disability). In addition, based on whether disabled conditions were inherited, we constructed outcome variables including whether having inherited disability and acquired disability.

The independent variables included maternal and paternal education. We treated maternal and paternal education as categorical variables in sample distribution and the prevalence of varying educational groups. In addition, we also treated maternal and paternal education as interval variables in logit regressions for two reasons. First, with the probability of child disability as the predicted variable in our regressions, the coefficients for years of schooling were easily interpreted as a rate of return and compared internationally [34]. Second, the effects of education on child disability with one degree of freedom allowed us to make cross-year comparisons. Following previous studies on schooling in China [34, 35], we extrapolated years of schooling from levels of attained education (illiteracy = 0; no formal education = 1; primary school = 6; middle school = 9; high school = 12; trade school = 13; community/technical college = 15; and college and graduate school = 17).

Control variables included the characteristics of children, parents, and their households. In detail, children’s control variables included their gender (male and female), age (0–14 years) and ethnicity (Han and minority). Parents’ control variables included their age at childbirth (15–59 years), employment status (unemployed or not), and disabled status (disabled or not). Household control variables included number of family members and residence (urban and rural). Province dummies (to ensure the consistency of two-wave data, Hainan and Guangdong Province in 2006 wave are both coded as Guangdong) were used to control provincial heterogeneity. Household income is excluded from controls because the 1987 survey did not have a measure of household income.

### Ethical approval

The surveys were conducted in all provinces by the Leading Group of the National Sample Survey on Disability and the National Bureau of Statistics. The 1987 and 2006 surveys were both approved by the China State Council and implemented within the legal framework governed by the Statistical Law of the People’s Republic of China. The children’s parents or carers signed the informed consent with interviewers to participate in the survey and clinical diagnosis.

### Statistical analysis

This study aimed to examine whether the prevalence of child disability was altered by accounting for parental education. To quantify this association, a reduced-form health equation was assumed to take the following form[36]:

$$H_{i}=f\left(x_{i},\;x_{c},\;x_{p},\;x_{h},\;\varepsilon_{i}\right)$$

Where *H*_*i*_ is the disabled status of the child; *x*_*i*_ is the parental education including maternal and paternal education; *x*_*c*_, *x*_*p*_ and *x*_*h*_ are vectors of children’s, parents’ and household-level characteristics, respectively; and *ε*_*i*_ is a composite error term of unobserved child, parents, household, and other heterogeneity.

The marginal effects of independent variables were calculated, and the final equation of estimation is expressed as the following:

$$Y_{ij}=\beta_{0}+\beta_{1}*MEDU_{ij}+\beta_{2}*FEDU_{ij}+\beta_{3}*X_{ij}+\beta_{4}*D_{j}+\varepsilon_{ij}$$

Where *MEDU*_*ij*_ and *FEDU*_*ij*_ refer to the years of maternal and paternal schooling of child i in Province j, respectively; *X*_*ij*_ represents all control variables; *D*_*j*_ is the dummy for province j; and *ε*_*ij*_ is the estimation error. When calculating marginal effects of maternal and paternal education, we controlled all covariates and province dummies. Binary logit regression models were used to examine the association of parental education with child disability. P value less than 0.05 was set as statistically significant. STATA 13.0 software for Windows (StataCorp, Texas, USA) was utilized for data analysis.

## Results

Table 1 shows the characteristics of participants. Overall, the prevalence of child disability was 2.6% in 1987 and 1.5% in 2006. The prevalence of inherited, acquired, physical and mental disability was 0.3%, 2.3%, 0.9% and 2.0%, respectively, in 1987, and 0.1%, 1.4%, 0.9% and 1.0%, respectively, in 2006. The average years of maternal and paternal schooling increased from roughly 4.1 and 6.7, respectively, in 1987, to 7.7 and 8.8, respectively, in 2006. Characteristics of covariates are also shown in Table 1.

**Table 1:** Characteristics of participants in 1987 and 2006.

Variables		1987 (n = 408096)	2006 (n = 356625)
Outcome variables			
Overall child disability, %		2.6	1.5
Inherited child disability, %		0.3	0.1
Acquired child disability, %		2.3	1.4
Physical child disability, %		0.9	0.9
Mental child disability, %		2.0	1.0
Independent variables			
Maternal education, mean(SD), years		4.1(4.1)	7.7(3.6)
Maternal education, %	Illiteracy	46.8	10.6
	Primary school	30.7	33.3
	Junior high school	16.3	41.4
	Senior high school or above	6.3	14.8
Paternal education, mean(SD), years		6.7(3.8)	8.8(3.1)
Paternal education, %	Illiteracy	17.7	3.2
	Primary school	39.3	27.0
	Junior high school	30.1	50.0
	Senior high school or above	12.9	19.8
Covariates			
Age of children, mean(SD), years		7.1(4.4)	8.0(4.2)
Sex of children, %	Male	51.8	54.0
	Female	48.2	46.0
Ethnicity of children, %	Han	88.6	84.4
	Others	11.4	15.6
Residence of children, %	Rural	75.3	71.4
	Urban	24.7	28.6
Family members, mean(SD), persons		5.3(1.7)	4.5(1.3)
Maternal unemployment, %	No	76.9	84.5
	Yes	23.1	15.5
Paternal unemployment, %	No	98.4	97.0
	Yes	1.6	3.0
Disabled mothers, %	No	97.0	97.5
	Yes	3.0	2.5
Disabled fathers, %	No	97.8	97.4
	Yes	2.2	2.6
Maternal age at childbirth, mean(SD), years		26.7(5.2)	26.1(4.7)
Paternal age at childbirth, mean(SD), years		29.4(6.0)	28.0(5.2)

Table 2 reports the prevalence of child disability by maternal and paternal education. Families with higher-level maternal and paternal education have lower prevalence of child disability in both years compared with those with lower-level maternal and paternal education. Analyses stratified by sex of children show similar results to overall analysis in all sample.

**Table 2:** Prevalence (%) of child disability in 1987 and 2006, by maternal and paternal education.

	All children		Male children		Female children	
	1987	2006	1987	2006	1987	2006
Maternal education						
Illiteracy	3.4	3.1	3.7	3.4	3.2	3.6
Primary school	2.4	1.8	2.5	1.9	2.2	2.8
Junior high school	1.3	1.2	1.5	1.3	1.2	1.7
Senior high school or above	1.0	0.8	1.2	0.9	0.9	1.3
Paternal education						
Illiteracy	4.1	4.0	4.6	4.2	2.8	3.7
Primary school	3.0	2.0	3.1	2.2	1.6	1.8
Junior high school	1.8	1.3	2.0	1.5	1.0	1.2
Senior high school or above	1.4	0.9	1.4	1.1	0.6	0.7

Table 3 presents the estimates on the association of maternal and paternal education with child disability. In the total sample, Model 1 and Model 2 show that in both surveys, the years of maternal and paternal schooling were each significantly associated with child disability, after controlling for all covariates this study used. With further consideration, after placing both parents’ schoolings into one equation, Model 3 demonstrates that maternal and paternal schooling were still significantly associated with child disability in both surveys, although the coefficients experienced reductions. In the subsample of male and female children, we found similar results to the total sample.

**Table 3:** Logit models for the association of maternal and paternal education with child disability in 1987 and 2006.

Sample		1987			2006		
		Model 1	Model 2	Model 3	Model 1	Model 2	Model 3
All children	Maternal education	-0.060*** (0.003)		-0.048*** (0.003)	-0.080*** (0.004)		-0.062*** (0.005)
	Paternal education		-0.051*** (0.003)	-0.036*** (0.003)		-0.079*** (0.005)	-0.048*** (0.006)
	Covariates	Yes	Yes	Yes	Yes	Yes	Yes
	N	408076	408076	408076	356625	356625	356625
Male children	Maternal education	-0.056*** (0.004)		-0.043*** (0.004)	-0.077*** (0.006)		-0.061*** (0.006)
	Paternal education		-0.056*** (0.004)	-0.042*** (0.004)		-0.075*** (0.007)	-0.044*** (0.007)
	Covariates	Yes	Yes	Yes	Yes	Yes	Yes
	N	211402	211402	211402	192639	192639	192639
Female children	Maternal education	-0.064*** (0.005)		-0.055*** (0.005)	-0.083*** (0.007)		-0.062*** (0.008)
	Paternal education		-0.046*** (0.004)	-0.029*** (0.005)		-0.085*** (0.008)	-0.054*** (0.009)
	Covariates	Yes	Yes	Yes	Yes	Yes	Yes
	N	196674	196674	196674	163986	163986	163986

Robust standard errors in parentheses;

* P < 0.05.

** P < 0.01.

*** P < 0.001.

All models controlled for all covariates listed in Table 1.

Table 4 shows the marginal effects of maternal and paternal education in the probability of child disability as predicted by Model 3 in Table 1. Maternal education was more important than paternal education in determining child disability, regardless of whether female, male, or all samples, due to the fact that all marginal effects of maternal education were higher than those of paternal education in both years. In addition, compared to 1987, 2006 saw a decrease in the marginal effects of either parent’s education on child disability. Specifically, in 1987, a one-year increase in maternal and paternal schooling led to an average decrease of 0.121% and 0.091% in the probability of child disability, but 19 years later, these figures had dwindled to 0.091% and 0.072%, respectively.

**Table 4:** Marginal effects of maternal and paternal education on predicted probabilities (%) of child disability.

	All children		Male children		Female children	
	1987	2006	1987	2006	1987	2006
Maternal education	-0.121 (0.008)	-0.091 (0.007)	-0.115 (0.012)	-0.098 (0.011)	-0.127 (0.011)	-0.082 (0.010)
Paternal education	-0.091 (0.008)	-0.072 (0.009)	-0.114 (0.011)	-0.072 (0.012)	-0.067 (0.011)	-0.071 (0.012)

Robust standard errors in parentheses;

All marginal effects were based on Model 3 in Table 3.

S2 Table shows robust checks of independent association of maternal and paternal education with child disability. To check whether there was an independent association between maternal education and child disability, we performed separate regressions by groups with varying paternal education. In the subsamples of paternal education of illiteracy and primary school as well as junior high school and above, we both found independent associations of maternal education and child disability in both years. Likewise, we also found independent associations of paternal education with child disability in both years.

S3 Table presents robust checks of association of maternal and paternal education with child disability using different outcome variables. We found consistent results on the association of each parent’s education and children’s inherited disability, acquired disability, physical disability and mental disability.

## Discussion

Since the implementation of the reform and opening up policy in China, adults’ completed educational attainment and children’s health status have both made great progress. Due to a lack of research focusing on the association between parental education and child disability in China, this study makes a number of contributions.

First, our sample size, including data from each province in China from 1987 to 2006, is much larger (with more than 760,000 observations) than that in other Chinese studies [27, 28]. This secures a higher degree of statistical power and considerable external validity, and therefore we have been able to obtain more precise estimates on the association between parental education and child disability. We found a significant association between education for each parent and child disability, while other research found a significant impact of only maternal education on child disability [37]. Our results also comport well with previous studies in other developing countries in Asia, such as Indonesia, Bangladesh and Pakistan, where they found the negative effects of each parent’s educational level on child disability [26, 36].

Second, we investigated the relative importance of maternal and paternal education on child disability, contributing to resolving this controversy issue based on two national surveys across almost 20 years. We found that maternal education was more important than paternal education in child disability in China in both surveys, which is consistent with earlier studies [37]. This may be because children develop a specific and enduring relationship with their primary caregivers [15], and mothers most often play the role of primary caregivers [38], based on the attachment theory [39], as well as Grossman’s theory of the division of labor [40]. The methodology of this study is different from that of prior studies, in that we used marginal effects to compare the relative importance of maternal and paternal education, whereas prior studies directly compared the regression coefficients of each parent [37].

Third, this study demonstrated whether the association of maternal and paternal education with child disability changed over time. In 1987–2006, the average years of maternal and paternal schooling increased from 4.1 and 6.7, respectively, to 7.7 and 8.8, respectively, while the prevalence of child disability decreased from 2.6% to 1.5%. The significant association between each parent’s education and child disability in both surveys shows that this relationship is robust over 19 years despite the change in values for each variable. This is also a unique finding in China compared to other developing regions where they only used one-year cross-sectional data [26, 36]. In addition, it is reasonable that the marginal effects (absolute values) of each parent’s education on child disability have declined over time, because of the principle of the diminishing marginal effects of education on health.

Finally, to check the robust association of maternal and paternal education with child disability, we performed estimations using subsamples of different education and different disability outcomes. Consequently, we found each parent’s education was independently associated with child disability. In addition, we found significant associations of both parents’ education with children’s inherited disability, acquired disability, physical disability and mental disability. These checks indicate our estimates on such association are robust.

While we do show benefits above, there are several limitations in this study. The first limitation is that we are unable to obtain a causal effect of parental education on child disability due to the cross-sectional design. In addition, the mechanism of the effect of parental education on child disability is unclear from the present study. If possible, further studies should take into account the potential pathways of this relationship, such as parental health knowledge as well as health care utilization [22]. Furthermore, due to a lack of data, we did not include income as a control variable in the regressions, which may influence our estimation results.

Despite these limitations, this study shows that it is important to appreciate the role of each parent’s education in the reduction of the prevalence of child disability in China. This implies that Chinese policymakers should not only consider strengthening health systems to provide better health services to children, but also should pay close attention to social determinants on child health, such as educational attainment among parents.

## Conclusion

The educational attainment of each parent was negatively associated with child disability in China. The education of mothers played a more critical role than that of fathers in child disability. In addition, we found downward trends in the marginal effects of parental education on child disability over 19 years.

## Supporting information

S1 Table The definitions and survey questions for different types of disabilities. (DOCX)

S2 Table Robust check of independent association of maternal and paternal education with child disability in 1987 and 2006, based on logit models. (DOCX)

S3 Table Robust check of association of maternal and paternal education with child disability in 1987 and 2006, by different outcomes. (DOCX)
