# Supplementary material for: The role of parental education in child disability in China from 1987 to 2006
Source: PLoS One. 2017 Oct 17;12(10):e0186623. doi: 10.1371/journal.pone.0186623 (PMC5645139; doi:10.1371/journal.pone.0186623)
Supplement: S1 Table — (DOCX) [file pone.0186623.s001.docx]

**S1** **Table.** The definitions and survey questions for diﬀerent types of disabilities

| Types | Definition | Questions in questionnaire |
| --- | --- | --- |
| Visual disability | Visual disability was defined as poor vision and/or constriction of visual fields (both eyes) from an uncorrectable cause, affecting daily life and social participation. | Do you or any of your family members has eyesight problem? |
| Hearing and  speech disability | Hearing disability was defined as permanent hearing loss of varying degrees from any cause or the inability to hear at all or to hear clearly any nearby sound or voice, which aﬀect daily life and social activities.  Speech disability was defined as any type of language disorder. Because successful treatment takes more than 1 year and the disability is generally present for more than 2 years, the patient cannot take part in normal language exchanges, which undermines his or her daily life and participation in social activities. | Do you or your family members have hearing or speech problems? |
| Motor disability | Motor disability was defined as a loss of motor function of varying degrees or to limitations in movements or activities resulting from deformed limbs or body paralysis (palsy) or from deformity caused by damage to the structure or function of those body parts involved in mobility. | Do you or any of your family members has difficulty in walking, standing, sitting, climbing upstairs, holding, writing, washing or dressing by hand in daily life? |
| Intellectual disability | Intellectual disability was defined as lower than normal intellectual ability and is accompanied by adaptive behavior disorders. This kind of disability results from impairment of the structure and functions of the nervous system, limits individual activity and participation, and requires all-round, extensive, limited, or intermittent support. | Do you or your family members have any difficulty studying? |
| Psychiatric disability | Psychiatric disability was defined as any psychiatric condition of >1-year duration, manifesting as a cognitive, affective, or behavioral disorder and affecting the daily life and social participation of the patient. | Are you or your family members forgetful? Or do you have difficulty concentrating? Or can you not control your moods? Or do you have strange behaviour that is out of the ordinary? Or are you addicted to alcohol or drugs? |
