# Supplementary material for: The role of parental education in child disability in China from 1987 to 2006
Source: PLoS One. 2017 Oct 17;12(10):e0186623. doi: 10.1371/journal.pone.0186623 (PMC5645139; doi:10.1371/journal.pone.0186623)
Supplement: S2 Table — (DOCX) [file pone.0186623.s002.docx]

**S2 Table.** Robust check of independent association of maternal and paternal education with child disability in 1987 and 2006, based on logit models

|  | Paternal education | | | | Maternal education | | | |
| --- | --- | --- | --- | --- | --- | --- | --- | --- |
|  | 1987 | | 2006 | | 1987 | | 2006 | |
|  | Illiteracy and primary school | Junior high school and above | Illiteracy and primary school | Junior high school and above | Illiteracy and primary school | Junior high school and above | Illiteracy and primary school | Junior high school and above |
| Maternal education | -0.051*** (0.004) | -0.049*** (0.005) | -0.070*** (0.007) | -0.070*** (0.006) | - | - | - | - |
| Paternal education | - | - | - | - | -0.043*** (0.003) | -0.038*** (0.010) | -0.065*** (0.007) | -0.060*** (0.009) |
| Covariates | Yes | Yes | Yes | Yes | Yes | Yes | Yes | Yes |
| N | 232420 | 175635 | 107751 | 248874 | 315987 | 92089 | 156428 | 200197 |

Robust standard errors in parentheses;

** P < 0.05, ** P < 0.01, *** P < 0.001;*

All models controlled for all covariates listed in Table 1.
