# Supplementary material for: The role of parental education in child disability in China from 1987 to 2006
Source: PLoS One. 2017 Oct 17;12(10):e0186623. doi: 10.1371/journal.pone.0186623 (PMC5645139; doi:10.1371/journal.pone.0186623)
Supplement: S3 Table — (DOCX) [file pone.0186623.s003.docx]

**S3 Table.** Robust check of association of maternal and paternal education with child disability in 1987 and 2006, by different outcomes

|  | Inherited disability | | Acquired disability | | Physical disability | | Mental disability | |
| --- | --- | --- | --- | --- | --- | --- | --- | --- |
|  | 1987 | 2006 | 1987 | 2006 | 1987 | 2006 | 1987 | 2006 |
| Maternal education | -0.071*** (0.010) | -0.100*** (0.017) | -0.045*** (0.003) | -0.059*** (0.005) | -0.023*** (0.005) | -0.051*** (0.006) | -0.057*** (0.004) | -0.067*** (0.006) |
| Paternal education | -0.065*** (0.009) | -0.042* (0.019) | -0.032*** (0.003) | -0.050*** (0.006) | -0.010* (0.005) | -0.039*** (0.007) | -0.044*** (0.003) | -0.059*** (0.007) |
| Covariates | Yes | Yes | Yes | Yes | Yes | Yes | Yes | Yes |
| N | 398669 | 347817 | 406805 | 356152 | 400939 | 354436 | 405504 | 354600 |

Robust standard errors in parentheses;

** P < 0.05, ** P < 0.01, *** P < 0.001;*

All models controlled for all covariates listed in Table 1.
